# Impact of postoperative radiotherapy for T3N0M0 esophageal cancer patients: A population‐based study

**DOI:** 10.1002/ctm2.143

**Published:** 2020-07-28

**Authors:** Yizhi Ge, Li Yin, Mingdian Tan, Weixing Dai, Yaqi Jiang, Li Chen, Xia He

**Affiliations:** ^1^ Department of Radiation Oncology The Affiliated Cancer Hospital of Nanjing Medical University (Jiangsu Cancer Hospital) and Jiangsu Institute of Cancer Research Nanjing Jiangsu China; ^2^ Asian Liver Center and Department of Surgery Stanford University School of Medicine Stanford University, Standford California; ^3^ Department of Colorectal Surgery Fudan University Shanghai Cancer Center Shanghai China; ^4^ Department of Oncology Northern Jiangsu People's Hospital Yangzhou China

Dear Editor,

The prognostic impact of postoperative radiotherapy (PORT) in esophageal cancer (EC) patients with negative lymph nodes has been investigated for decades. However, the data remain inconclusive and histological distinctions have not been studied. Among EC, adenocarcinoma (AC) and squamous cell carcinoma (SCC) are the two most common histological types,^1^ but they differ in epidemiological and molecular features.^2^ How histological types alter therapeutic responses to PORT are unknown. This study assessed the relationship between PORT and histological types in pT3N0M0 EC patients. From the Surveillance, Epidemiology, and End Results (SEER) database, we selected 451 pT3N0M0 EC patients for our analysis. Among them, 348 (77.2%) cases had surgery alone and 103 (22.8%) received PORT. The detailed information of patients’ features is shown in Table S1. Significantly higher proportion of cases with chemotherapy treatment was observed in patients who received PORT (Table S1).

We used Kaplan‐Meier survival analysis to analyze the impact of PORT on overall survival (OS), and cumulative incidence function (CIF) for cancer‐specific survival (CSS) analysis. The five‐year OS rate was 27.9% for the whole EC patients (Figure 1A), and the median survival was 25 months (Table S2). In the surgery group, the 5‐year OS was 27.1% while it remained 30.5% for patients who received PORT. The median survival was increased by 3 months in the PORT group as compared with the surgery group (Table S2). However, the prognosis between AC and SCC patients was not statistically different in OS (*P *= .220) (Figure 1B) and CSS (*P *= .318) (Figure 1C). Interestingly, the multivariable Cox analysis indicated the PORT was an independent prognostic factor in predicting poor CSS (*P *< .001) (Table S3). This indicated that PORT has no significant survival benefit, which is consistent with some previous research.^3^, ^4^

**FIGURE 1:**
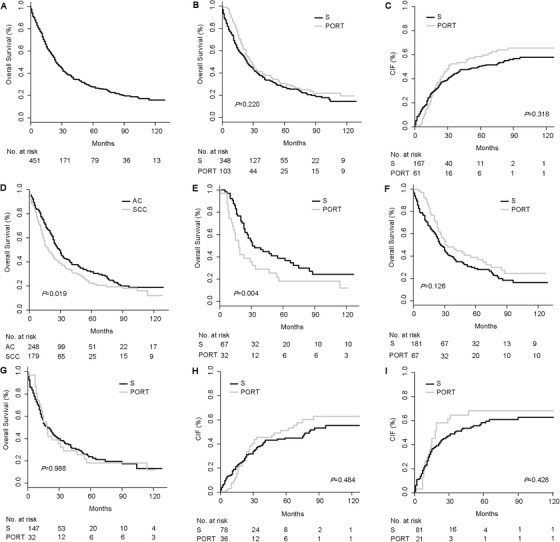
Survival and cumulative incidence function analysis of esophageal cancer patients in pT3N0M0 before propensity score matching analysis. A, Overall survival of the whole EC patients. B, Survival of patients stratified by PORT status. C, CIF of overall patients. D, Survival of patients stratified by histological types. E, Survival of PORT subgroup stratified by histological types. F, Survival of AC patients stratified by PORT status. G, Survival of SCC patients stratified by PORT status. H, CIF of AC patients. I, CIF of SCC patients. Abbreviation: AC, Adenocarcinoma; CIF, cumulative incidence function; EC, esophageal cancer; No., numbers; PORT, postoperative radiotherapy; S, surgery; SCC, squamous cell carcinoma

We assumed the prognostic value of PORT was different between histological types. The multivariable logistic regression analysis indicated that chemotherapy was associated with significantly greater odds of receiving PORT, while 1‐10 and 11‐20 pathologically examined lymph nodes (eLNs) were associated with lower odds in the AC group (Table S4). In the SCC group, cancer occurred in the middle third of the esophagus, with >20 eLNs and having chemotherapy were associated with significantly greater odds of receiving PORT, while tumor size of 21–40 and >80 mm were associated with lower odds of receiving PORT (Table S4).

The 5‐year OS rate was 31.7% and 22.4%, and the median survival was 29 months and 18 months, respectively, in the AC and SCC groups. OS difference and the impact of PORT were statistically significant between the two groups (*P *= .019 and .004) (Figure 1D,E). Consistent with the entire cohort, PORT did not statistically improve the OS of AC (*P *= .126) or SCC patients (*P *= .988) (Figure 1F,G), neither did CSS in AC (*P *= .484) (Figure 1H) or SCC group (*P *= .428) (Figure 1I). In EC‐specific mortality multivariable analysis, PORT maintained to be independently associated with decreased CSS (*P *= .010) in the AC group (Table S5). Instead, PORT was not a prognostic factor for both OS (*P *= .566) and CSS (*P *= .320) in the SCC group (Table S6). These results revealed the distinct influence of PORT on prognosis in patients with AC or SCC. Taken together, we concluded that PORT could independently predict poor prognosis in AC patients, but not for the SCC group.

Before conducting propensity score matching (PSM) analysis, CIF analysis did not indicate the PORT's prognostic significance. This may be resulted from potential bias of the data. Considering the inconsistence of result from univariate and multivariate CSS analysis, we performed PSM analysis to adjust the variables and confirmed the above results. After PSM, 66 matched patients were identified and no difference in clinical characteristics was found between the two groups (Table 1). The 5‐year OS rate was 20.8% (Figure 2A), and the median survival was 25 months (Table S1) in the whole cohort. Consistent with results before PSM, the OS and the impact of PORT were different between the two groups (*P *= .004 and .017) (Figure 2B,C). Moreover, PORT was not notably associated with OS in the entire cohort (*P *= .613) (Figure 2D) or AC (*P *= .937) (Figure 2E) and SCC group (*P *= .764) (Figure 2F). Instead, patients with PORT showed significantly worse CSS than patients without PORT in the overall patients (*P *= .003) (Figure 2G). Further subgroup analysis based on histology confirmed that PORT was independently related to poor CSS in the AC group (*P *= .046) (Figure 2H) but not in SCC patients (*P *= .139) (Figure 2I). These results confirmed the adverse impact of PORT on CSS in AC patients. Collectively, given that PORT could worsen prognosis, we do not recommend PORT for pT3N0M0 EC patients, regardless of histological types (AC or SCC).

**FIGURE 2:**
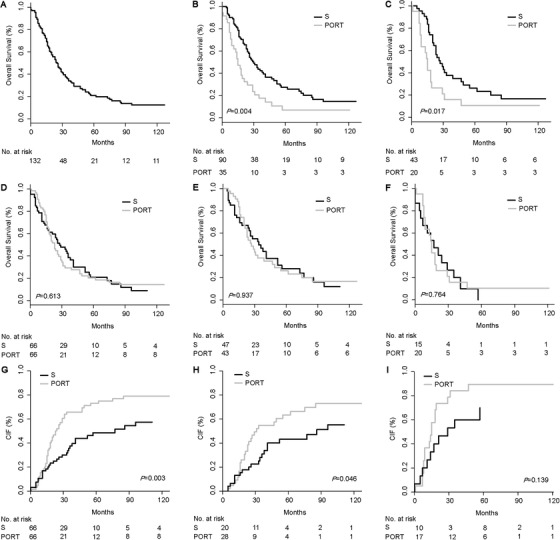
Survival and cumulative incidence function of esophageal cancer patients in pT3N0M0 after propensity score matching analysis. A, Overall survival of the whole EC patients. B, Survival of patients stratified by histological types. C, Survival of PORT subgroup stratified by histological types. D, Survival of patients stratified by PORT status. E, Survival of AC patients stratified by PORT status. F, Survival of SCC patients stratified by PORT status. G, CIF of overall patients. H, CIF of AC patients. I, CIF of SCC patients. Abbreviation: AC, adenocarcinoma; CIF, cumulative incidence function; EC, esophageal cancer; No., numbers; PORT, postoperative radiotherapy; S, surgery SCC, squamous cell carcinoma

In conclusion, the prognostic value of PORT was distinct in AC and SCC patients. PORT is not recommended for pT3N0M0 EC patients for lack of survival benefit. These findings may assist oncologists and patients in making treatment decisions. In light of updated radiotherapy techniques and treatment planning, further clinical research is needed to comprehensively evaluate PORT's value.

## CONFLICT OF INTEREST

The authors declare that there is no conflict of interest.

## AUTHORS CONTRIBUTION

Yizhi Ge and Li Yin performed research design, data collection, interpretation and analysis, and manuscript drafting. They contribute equally to this work. Mingdian Tan, Weixing Dai, and Yaqi Jiang are associated with data collection, analysis, interpretation, and critical revision for this manuscript. Xia He and Li Chen performed research design, data analysis, results interpretation, paper writing, and critical revision of this manuscript. All authors approved the final version and agreed to be accountable for all aspects of the work.

## Supporting information

Supporting Information

## Data Availability

The data that support the findings of this study are available in the public domain: https://seer.cancer.gov/.
