# Supplementary material for: Impact of postoperative radiotherapy for T3N0M0 esophageal cancer patients: A population‐based study
Source: Clin Transl Med. 2020 Jul 28;10(3):e143. doi: 10.1002/ctm2.143 (PMC7418799; doi:10.1002/ctm2.143)
Supplement: Supplementary file 1 — Supporting Information [file CTM2-10-e143-s001.pdf]

Supplementary Tables:

Supplementary Table S1. Characteristics of pT3N0M0 Esophageal Cancer Patients

Supplementary Table S2. Median Survival of Esophagus Cancer Patients Stratified by Postoperative Radiotherapy

Supplementary Table S3. Multivariable Cox Regression for All-cause and Esophagus Cancer-Specific Mortality before Propensity Score Matching Analysis

Supplementary Table S4. Multivariable Logistic Regression for the Postoperative Radiotherapy in Esophageal Cancer Patients before Propensity Score Matching Analysis

Supplementary Table S5. Multivariable Cox Regression for All-cause and Cancer-Specific Mortality in AC Patients before Propensity Score Matching Analysis

Supplementary Table S6. Multivariable Cox Regression for All-cause and Cancer-Specific Mortality in SCC Patients before Propensity Score Matching Analysis

Table S1. Characteristics of pT3N0M0 Esophageal Cancer Patients

	Before PSM				After PSM			
**Variable**	**Total Patients No. (%)**	**Surgery No. (%)**	**PORT No. (%)**	***P* Value[c]**	**Total Patients No. (%)**	**Surgery No. (%)**	**PORT No. (%)**	***P* Value[c]**
**Total**	451 (100)	348 (77.2)	103 (22.8)		132 (100)	66 (50.0)	66 (50.0)	
**Age[a]**				0.005				0.170
<65	199 (44.1)	148 (42.5)	51 (49.5)		59 (44.7)	26 (39.4)	33 (50.0)	
65-80	212 (47.0)	161 (46.3)	51 (49.5)		67 (50.8)	35 (53.0)	32 (48.5)	
>80	40 (8.9)	39 (11.2)	1 (1.0)		6 (4.5)	5 (7.6)	1 (1.5)	
**Sex**				0.076				0.819
Male	333 (73.8)	250 (71.8)	83 (80.6)		109 (82.6)	54 (81.8)	55 (83.3)	
Female	118 (26.2)	98 (28.2)	20 (19.4)		23 (17.4)	12 (18.2)	11 (16.7)	
**Race**				0.869				0.051
White	369 (81.8)	284 (81.6)	85 (82.5)		111 (84.1)	58 (87.9)	53 (80.3)	
Black	46 (10.2)	37 (10.6)	9 (8.7)		15 (11.4)	8 (12.1)	7 (10.6)	
Others[b]	35 (7.8)	27 (7.8)	8 (7.8)		6 (4.5)	0 (0.0)	6 (9.1)	
Unknown	1 (0.2)	0 (0.0)	1 (1.0)		0 (0.0)	0 (0.0)	0 (0.0)	
**Marital Status**				0.290				0.329
Unmarried	75 (16.6)	61 (17.5)	14 (13.6)		20 (15.2)	8 (12.1)	12 (18.2)	
Married	353 (78.3)	267 (76.7)	86 (83.5)		106 (80.3)	55 (83.3)	51 (77.3)	
Unknown	23 (5.1)	20 (5.7)	3 (2.9)		6 (4.5)	3 (4.5)	3 (4.5)	
**Insurance Status**				1.000				1.000
Uninsured	7 (1.6)	6 (1.7)	1 (1.0)		1 (0.8)	0 (0.0)	1 (1.5)	
Insured	264 (58.5)	207 (59.5)	57 (55.3)		79 (59.8)	40 (60.6)	39 (59.1)	
Unknown	180 (39.9)	135 (38.8)	45 (43.7)		52 (39.4)	26 (39.4)	26 (39.4)	
**Grade**				0.395				0.918
I	29 (6.4)	21 (6.0)	8 (7.8)		8 (6.1)	4 (6.1)	4 (6.1)	
II	196 (43.5)	158 (45.4)	38 (36.9)		50 (37.9)	26 (39.4)	24 (36.4)	
III	205 (45.5)	154 (44.3)	51 (49.5)		67 (50.8)	32 (48.5)	35 (53.0)	
IV	4 (0.9)	4 (1.1)	0 (0.0)		0 (1.4)	0 (0.0)	0 (0.0)	
Unknown	17 (3.8)	11 (3.2)	6 (5.8)		7 (5.3)	4 (6.1)	3 (4.5)	
**Primary Site of Esophagus**				0.080				0.070
Upper	33 (7.3)	31 (8.9)	2 (1.9)		2 (1.5)	0 (0.0)	2 (3.0)	
Middle	69 (15.3)	53 (15.2)	16 (15.5)		16 (12.1)	5 (7.6)	11 (16.7)	
Lower	299 (66.3)	231 (66.4)	68 (66.0)		101 (76.5)	55 (83.3)	46 (69.7)	
Unknown	50 (11.1)	33 (9.5)	17 (16.5)		13 (9.8)	6 (9.1)	7 (10.6)	
**Histological Types**				0.690				0.679
AC	248 (55.0)	181 (52.1)	67 (65.0)		90 (68.2)	47 (71.2)	43 (65.2)	
SCC	179 (39.7)	147 (42.2)	32 (31.1)		35 (26.5)	15 (22.7)	20 (30.3)	

Others	23 (5.1)	19 (5.5)	4 (3.9)		7 (5.3)	4 (6.1)	3 (4.5)	
Unknown	1 (0.2)	1 (0.3)	0 (0.0)		0 (0.0)	0 (0.0)	0 (0.0)	
**Tumor Size, mm**				0.665				0.168
0-20	42 (9.3)	31 (8.9)	11 (10.7)		11 (8.3)	8 (12.1)	3 (4.5)	
21-40	202 (44.8)	163 (46.8)	39 (37.9)		54 (40.9)	30 (45.5)	24 (36.4)	
41-60	107 (23.7)	84 (24.1)	23 (22.3)		28 (21.2)	10 (15.2)	18 (27.3)	
61-80	38(8.4)	28 (8.0)	10 (9.7)		14 (10.6)	6 (9.1)	8 (12.1)	
>80	22 (4.9)	16 (4.6)	6 (5.8)		4 (3.0)	1 (1.5)	3 (4.5)	
Unknown	40 (8.9)	26 (7.5)	14 (13.6)		21 (15.9)	11 (16.7)	10 (15.2)	
**Examined LNs, No.**				0.226				0.083
0	26 (5.8)	17 (4.9)	9 (8.7)		8 (6.1)	3 (4.5)	5 (7.6)	
1-10	197 (43.7)	155 (44.5)	42 (40.8)		59 (44.7)	32 (48.5)	27 (40.9)	
11-20	148 (32.8)	119 (34.2)	29 (28.2)		44 (33.3)	26 (39.4)	18 (27.3)	
>20	71 (15.7)	51 (14.7)	20 (19.4)		19 (14.4)	5 (7.6)	14 (21.2)	
Unknown	9 (2.0)	6 (1.7)	3 (2.9)		2 (1.5)	0 (0.0)	2 (3.0)	
**Chemotherapy**				<0.001				1.000
No/Unknown	281(62.3)	258 (74.1)	23 (22.3)		46 (34.8)	23 (34.8)	23 (34.8)	
Yes	170 (37.7)	90 (25.9)	80 (77.7)		86 (65.2)	43 (65.2)	43 (65.2)	

Abbreviations: pT3N0M0, pathologically T3N0M0; PSM, propensity score matching; PORT, postoperative radiotherapy; AC, adenocarcinoma; SCC, squamous cell carcinoma; LNs, lymph nodes; No., numbers; NA, not available or not applicable.

[a] Age at diagnosis.

[b] Others include Asian or Pacific Islander and American Indian/Alaska Native.

[c] Chi-square test or Fisher's exact test were applied. This analysis does not include the subsets with “Unknown”.

Table S2. Median Survival of Esophagus Cancer Patients Stratified by Postoperative Radiotherapy

PORT	Median Survival. mo. (IQR)			Median Survival. mo. (IQR)		
	Before PSM			After PSM		
	All	AC	SCC	All	AC	SCC
No	24 (9-73)	26 (11-76)	19 (8-57)	28 (11-56)	34 (13-73)	17 (5-35)
Yes	27 (15-85)	31 (20-88)	18 (9-54)	23 (15-47)	27 (17-62)	15 (8-29)
All	25 (11-74)	29 (14-77)	18 (8-56)	25 (13-52)	30 (16-73)	15 (8-29)

Abbreviations: PORT, postoperative radiotherapy; mo., months; IQR, interquartile range; PSM, Propensity score matching; AC, adenocarcinoma; SCC, squamous cell carcinoma.

Table S3. Multivariable Cox Regression for All-cause and Esophagus Cancer-Specific Mortality before Propensity Score Matching Analysis

Variable	All-Cause Mortality (n= 451)		Esophagus Cancer–Specific Mortality[c] (n=221)	
	HR (95% CI)	*P* Value	HR (95% CI)	*P* Value
**Age[a]**				
<65	1 [Reference]	NA	1 [Reference]	NA
65-80	1.283 (0.996-1.652)	0.053	1.093 (0.817-1.461)	0.550
>80	1.776 (1.173-2.689)	0.007	1.046 (0.622-1.759)	0.860
**Sex**				
Male	1 [Reference]	NA	1 [Reference]	NA
Female	0.701 (0.526-0.933)	0.015	0.813 (0.580-1.139)	0.230
**Race**				
White	1 [Reference]	NA	1 [Reference]	NA
Black	1.119 (0.743-1.684)	0.590	1.275 (0.755-2.152)	0.360
Others	0.980 (0.615-1.562)	0.932	1.407 (0.827-2.396)	0.210
Unknown[b]	--	NA	--	NA
**Marital status**				
Unmarried	1 [Reference]	NA	1 [Reference]	NA
Married	0.974 (0.700-1.356)	0.877	1.326 (0.868-2.025)	0.190
Unknown	1.206 (0.664-2.188)	0.538	1.393 (0.631-3.073)	0.410
**Insurance status**				
Uninsured	1 [Reference]	NA	1 [Reference]	NA
Insured	0.849 (0.264-2.737)	0.784	1.388 (0.311-6.194)	0.670
Unknown	0.895 (0.278-2.881)	0.852	1.144 (0.255-5.131)	0.860
**Grade**				
I	1 [Reference]	NA	1 [Reference]	NA
II	1.521 (0.826-2.801)	0.178	1.706 (0.742-3.923)	0.210
III	1.989 (1.078-3.670)	0.028	1.904 (0.822-4.411)	0.130
IV	3.049 (0.641-14.500)	0.161	2.284 (0.147-35.602)	0.560
Unknown	1.694 (0.687-4.176)	0.252	1.375 (0.404-4.679)	0.610
**Primary Site of Esophagus**				
Upper	1 [Reference]	NA	1 [Reference]	NA
Middle	0.963 (0.572-1.622)	0.889	1.581 (0.820-3.050)	0.170
Lower	0.627 (0.375-1.050)	0.076	1.111 (0.568-2.171)	0.760
Unknown	0.811 (0.453-1.451)	0.480	1.140 (0.541-2.400)	0.730
**Histological Types**				
AC	1 [Reference]	NA	1 [Reference]	NA
SCC	1.064 (0.774-1.461)	0.704	1.282 (0.871-1.888)	0.210

Others	0.922 (0.532-1.599)	0.774	0.887 (0.445-1.770)	0.730
Unknown[b]	--	NA	--	NA
**PORT**				
No	1 [Reference]	NA	1 [Reference]	NA
Yes	1.160 (0.845-1.593)	0.359	1.807 (1.295-2.522)	<0.001
**Tumor Size**				
0-20	1 [Reference]	NA	1 [Reference]	NA
21-40	1.973 (1.261-3.087)	0.003	1.545 (0.936-2.550)	0.089
41-60	2.118 (1.313-3.416)	0.002	1.589 (0.928-2.721)	0.091
61-80	1.524 (0.825-2.816)	0.179	1.278 (0.648-2.521)	0.480
>80	1.715 (0.809-3.636)	0.160	1.201 (0.411-3.508)	0.740
Unknown	2.399 (1.346-4.276)	0.003	1.927 (1.004-3.697)	0.049
**Examined LNs, No.**				
0	1 [Reference]	NA	1 [Reference]	NA
1-10	0.878 (0.545-1.415)	0.593	1.186 (0.626-2.245)	0.600
11-20	0.667 (0.404-1.103)	0.114	0.887 (0.453-1.737)	0.730
>20	0.570 (0.327-0.994)	0.048	0.742 (0.357-1.541)	0.420
Unknown	0.829 (0.323-2.130)	0.697	1.288 (0.383-4.327)	0.680
**Chemotherapy**				
No/Unknown	1 [Reference]	NA	1 [Reference]	NA
Yes	0.566 (0.417-0.768)	<0.001	0.511 (0.363-0.720)	<0.001

Abbreviations: AC, adenocarcinoma; SCC, squamous cell carcinoma; PORT, postoperative radiation therapy; LNs, lymph nodes; NA, not available or not applicable.

[a] Age at diagnosis

[b] Only one case in this subgroup, so we excluded them in this analysis.

[c] Thirteen cases with 0 survival months were excluded in this analysis.

Table S4. Multivariable Logistic Regression for the Postoperative Radiotherapy in Esophageal Cancer Patients before Propensity Score Matching Analysis

Variable	Among Entire Esophagus Patients (n= 451)		Among AC Patients (n= 248)		Among SCC Patients (n= 179)	
	OR (95% CI)	*P* Value	OR (95% CI)	*P* Value	OR (95% CI)	*P* Value
**Age**[a]						
<65	1 [Reference]	NA	1 [Reference]	NA	1 [Reference]	NA
65-80	1.455 (0.831-2.547)	0.189	1.190 (0.576-2.461)	0.638	0.950 (0.222-4.068)	0.945
>80	0.173 (0.020-1.470)	0.108	0.204 (0.022-1.888)	0.161	0.000 (0.000-NA)	0.998
**Sex**						
Male	1 [Reference]	NA	1 [Reference]	NA	1 [Reference]	NA
Female	0.625 (0.314-1.245)	0.181	0.704 (0.237-2.092)	0.527	2.044 (0.467-8.953)	0.343
**Race**						
White	1 [Reference]	NA	1 [Reference]	NA	1 [Reference]	NA
Black	0.682 (0.244-1.903)	0.465	0.271 (0.024-3.115)	0.295	0.906 (0.148-5.559)	0.915
Others	1.631 (0.583-4.567)	0.352	0.995 (0.185-5.348)	0.995	4.566 (0.639-32.613)	0.130
Unknown	--	NA	--[b]	NA	--[c]	NA
**Marital Status**						
Unmarried	1 [Reference]	NA	1 [Reference]	NA	1 [Reference]	NA
Married	0.963 (0.438-2.119)	0.926	0.637 (0.221-1.835)	0.403	4.426 (0.446-43.921)	0.204
Unknown	0.386 (0.077-1.941)	0.248	0.186 (0.017-2.063)	0.171	0.000 (0.000-NA)	0.999
**Insurance Status**						
Uninsured	1 [Reference]	NA	1 [Reference]	NA	1 [Reference]	NA
Insured	0.508 (0.043-6.035)	0.592	1000+ (0.000-NA)	0.999	0.340 (0.008-13.723)	0.567
Unknown	0.825 (0.070-9.764)	0.879	1000+ (0.000-NA)	0.999	0.274 (0.008-9.989)	0.481
**Grade**						
I	1 [Reference]	NA	1 [Reference]	NA	1 [Reference]	NA
II	0.436 (0.131-1.454)	0.177	0.245 (0.039-1.547)	0.135	2.571 (0.196-33.695)	0.472
III	0.588 (0.180-1.923)	0.380	0.466 (0.078-2.762)	0.400	2.579 (0.214-31.088)	0.456
IV	0.000 (0.000-NA)	0.999	0.000 (0.000-NA)	1.000	0.000 (0.000-NA)	0.999
Unknown	0.561 (0.110-2.852)	0.486	0.448 (0.049-4.104)	0.478	0.448 (0.049-4.104)	0.478
**Primary Site of Esophagus**						
Upper	1 [Reference]	NA	1 [Reference]	NA	1 [Reference]	NA
Middle	4.220 (0.749-23.775)	0.103	1000+ (0.000-NA)	1.000	16.761 (1.521-184.746)	0.021
Lower	1.875 (0.343-10.246)	0.468	1000+ (0.000-NA)	1.000	1.659 (0.163-16.897)	0.669
Unknown	5.983 (0.993-36.058)	0.051	1000+ (0.000-NA)	1.000	165.375 (8.706-1000+)	0.001
**Histological Types**						
AC	1 [Reference]	NA	--	NA	--	NA
SCC	0.940 (0.446-1.985)	0.872	--	NA	--	NA
Others	0.899 (0.225-3.584)	0.880	--	NA	--	NA

Unknown[b]	--	NA	--	NA	--	NA
**Tumor Size, mm**						
0-20	1 [Reference]	NA	1 [Reference]	NA	1 [Reference]	NA
21-40	0.707 (0.272-1.843)	0.478	1.553 (0.419-5.752)	0.510	0.018 (0.001-0.301)	0.005
41-60	0.964 (0.352-2.644)	0.943	1.256 (0.300-5.262)	0.756	0.140 (0.014-1.359)	0.090
61-80	0.828 (0.245-2.798)	0.761	1.693 (0.333-8.600)	0.526	0.093 (0.003-2.581)	0.161
>80	0.671 (0.153-2.933)	0.596	1.100 (0.117-10.323)	0.933	0.020 (0.000-0.819)	0.039
Unknown	0.668 (0.208-2.152)	0.499	1.481 (0.335-6.550)	0.605	0.000 (0.000-NA)	0.998
**Examined LNs, No.**						
0	1 [Reference]	NA	1 [Reference]	NA	1 [Reference]	NA
1-10	0.449 (0.156-1.299)	0.140	0.179 (0.042-0.774)	0.021	8.878 (0.325-242.560)	0.196
11-20	0.289 (0.094-0.885)	0.030	0.125 (0.027-0.570)	0.007	8.366 (0.287-244.266)	0.217
>20	0.934 (0.286-3.051)	0.910	0.407 (0.081-2.049)	0.276	88.713 (2.324-1000+)	0.016
Unknown	0.758 (0.112-5.145)	0.776	0.054 (0.004-0.818)	0.035	1000+ (0.000-NA)	0.998
**Chemotherapy**						
No/Unknown	1 [Reference]	NA	1 [Reference]	NA	1 [Reference]	NA
Yes	12.684 (6.652-24.184)	<0.001	12.028 (4.781-30.262)	<0.001	64.281 (9.911-416.930)	<0.001

Abbreviations: AC, adenocarcinoma; SCC, squamous cell carcinoma; LNs, lymph nodes; No., numbers; NA, not available or not applicable.

[a] Age at diagnosis.

[b] Only one case in AC subset, so we excluded them in this analysis.

[c] No cases in this subset in SCC patients, so we excluded them in this analysis.

Table S5. Multivariable Cox Regression for All-cause and Cancer-Specific Mortality in AC Patients before Propensity Score Matching Analysis

Variable	All-Cause Mortality		Esophagus Cancer–Specific Mortality[b]	
	HR (95% CI)	*P* Value	HR (95% CI)	*P* Value
**Age[a]**				
<65	1 [Reference]	NA	1 [Reference]	NA
65-80	1.343 (0.932-1.934)	0.113	0.889 (0.579-1.363)	0.590
>80	2.503 (1.455-4.307)	0.001	1.032 (0.490-2.173)	0.930
**Sex**				
Male	1 [Reference]	NA	1 [Reference]	NA
Female	0.774 (0.463-1.296)	0.330	0.839 (0.481-1.463)	0.540
**Race**				
White	1 [Reference]	NA	1 [Reference]	NA
Black	0.445 (0.130-1.526)	0.198	0.000 (0.000-0.000)	<0.001
Others	1.059 (0.391-2.871)	0.910	2.290 (0.887-5.911)	0.087
Unknown[c]	--	NA	--	NA
**Marital status**				
Unmarried	1 [Reference]	NA	1 [Reference]	NA
Married	0.698 (0.419-1.164)	0.168	1.318 (0.661-2.629)	0.430
Unknown	1.835 (0.761-4.428)	0.177	2.264 (0.719-7.133)	0.160
**Insurance status**				
Uninsured	1 [Reference]	NA	1 [Reference]	NA
Insured	1.314 (0.172-10.049)	0.792	1.269 (0.132-12.237)	0.840
Unknown	1.406 (0.182-10.844)	0.744	0.760 (0.078-7.409)	0.810
**Grade**				
I	1 [Reference]	NA	1 [Reference]	NA
II	0.948 (0.383-2.344)	0.908	1.135 (0.317-4.066)	0.850
III	1.532 (0.632-3.712)	0.345	1.665 (0.482-5.749)	0.420
IV	1.216 (0.133-11.140)	0.863	0.000 (0.000-0.000)	<0.001
Unknown	1.944 (0.611-6.187)	0.260	1.436 (0.291-7.084)	0.660
**Primary Site of Esophagus**				
Upper	1 [Reference]	NA	1 [Reference]	NA
Middle	0.169 (0.014-1.991)	0.158	0.168 (0.033-0.856)	0.032
Lower	0.107 (0.010-1.143)	0.064	0.135 (0.034-0.525)	0.004
Unknown	0.084 (0.007-0.975)	0.048	0.054 (0.010-0.279)	<0.001
**PORT**				
No	1 [Reference]	NA	1 [Reference]	NA
Yes	1.019 (0.664-1.563)	0.933	1.858 (1.163-2.968)	0.010
**Tumor Size**				

0-20	1 [Reference]	NA	1 [Reference]	NA
21-40	3.360 (1.592-7.092)	0.001	2.436 (0.878-6.756)	0.087
41-60	3.770 (1.716-8.282)	0.001	3.090 (1.013-9.425)	0.047
61-80	2.012 (0.812-4.988)	0.131	1.975 (0.659-5.921)	0.220
>80	1.345 (0.265-6.831)	0.721	0.000 (0.000-0.000)	<0.001
Unknown	3.890 (1.655-9.142)	0.002	3.084 (1.013-9.384)	0.047
**Examined LNs, No.**				
0	1 [Reference]	NA	1 [Reference]	NA
1-10	0.987 (0.464-2.100)	0.973	1.704 (0.624-4.651)	0.300
11-20	0.974 (0.457-2.076)	0.945	1.716 (0.605-4.868)	0.310
>20	0.811 (0.350-1.879)	0.624	1.365 (0.430-4.329)	0.600
Unknown	0.864 (0.265-2.816)	0.809	1.633 (0.297-8.975)	0.570
**Chemotherapy**				
No/Unknown	1 [Reference]	NA	1 [Reference]	NA
Yes	0.526 (0.352-0.787)	0.002	0.456 (0.286-0.726)	<0.001

Abbreviations: AC, adenocarcinoma; PORT, postoperative radiation therapy; LNs, lymph nodes; NA, not available or not applicable.

[a] Age at diagnosis.

[b] Eight cases with 0 survival months were excluded in this analysis.

[c] Only one case in this subgroup, so we excluded them in this analysis.

Table S6. Multivariable Cox Regression for All-cause and Cancer-Specific Mortality in SCC Patients before Propensity Score Matching Analysis

Variable	All-Cause Mortality		Esophagus Cancer–Specific Mortality[b]	
	HR (95% CI)	*P* Value	HR (95% CI)	*P* Value
**Age**[a]				
< 65	1 [Reference]	NA	1 [Reference]	NA
65-80	1.344 (0.884-2.041)	0.166	1.227 (0.739-2.039)	0.430
>80	1.166 (0.576-2.360)	0.669	1.092 (0.487-2.446)	0.830
**Sex**				
Male	1 [Reference]	NA	1 [Reference]	NA
Female	0.567 (0.382-0.840)	0.005	0.659 (0.413-1.053)	0.081
**Race**				
White	1 [Reference]	NA	1 [Reference]	NA
Black	1.032 (0.627-1.701)	0.900	1.201 (0.651-2.218)	0.560
Others	1.244 (0.644-2.402)	0.515	1.290 (0.612-2.719)	0.500
**Marital status**				
Unmarried	1 [Reference]	NA	1 [Reference]	NA
Married	1.148 (0.677-1.945)	0.609	1.128 (0.616-2.067)	0.700
Unknown	0.676 (0.241-1.893)	0.456	1.052 (0.326-3.390)	0.930
**Insurance status**				
Uninsured	1 [Reference]	NA	1 [Reference]	NA
Insured	0.499 (0.112-2.229)	0.363	1.718 (0.169-17.472)	0.650
Unknown	0.553 (0.125-2.434)	0.433	1.902 (0.185-19.611)	0.590
**Grade**				
I	1 [Reference]	NA	1 [Reference]	NA
II	2.624 (0.954-7.222)	0.062	2.113 (0.578-7.731)	0.260
III	3.067 (1.101-8.547)	0.032	1.938 (0.534-7.025)	0.310
IV[c]	-	NA	-	NA
Unknown[d]	-	NA	-	NA
**Primary Site of Esophagus**				
Upper	1 [Reference]	NA	1 [Reference]	NA
Middle	0.864 (0.461-1.617)	0.647	1.975 (0.927-4.205)	0.078
Lower	0.669 (0.371-1.209)	0.183	1.166 (0.531-2.557)	0.700
Unknown	1.012 (0.505-2.029)	0.972	1.869 (0.782-4.466)	0.160
**PORT**				
No	1 [Reference]	NA	1 [Reference]	NA
Yes	1.192 (0.654-2.170)	0.566	1.412 (0.714-2.792)	0.320
**Tumor Size**				
0-20	1 [Reference]	NA	1 [Reference]	NA

21-40	1.602 (0.829-3.095)	0.161	1.453 (0.686-3.078)	0.330
41-60	1.487 (0.751-2.945)	0.255	1.477 (0.664-3.283)	0.340
61-80	1.666 (0.591-4.696)	0.334	1.053 (0.294-3.774)	0.940
>80	1.188 (0.421-3.354)	0.745	1.145 (0.240-5.458)	0.860
Unknown	1.478 (0.512-4.264)	0.470	2.202 (0.777-6.245)	0.140
**Examined LNs, No.**				
0	1 [Reference]	NA	1 [Reference]	NA
1-10	0.682 (0.328-1.421)	0.307	0.794 (0.340-1.854)	0.590
11-20	0.428 (0.195-0.938)	0.034	0.507 (0.200-1.281)	0.150
>20	0.357 (0.146-0.871)	0.024	0.465 (0.169-1.282)	0.140
Unknown	0.960 (0.082-11.169)	0.974	1.963 (0.342-11.272)	0.450
**Chemotherapy**				
No/Unknown	1 [Reference]	NA	1 [Reference]	NA
Yes	0.554 (0.319-0.963)	0.036	0.491 (0.249-0.969)	0.040

Abbreviations: SCC, squamous cell carcinoma; PORT, postoperative radiation therapy; LNs, lymph nodes; NA, not available or not applicable.

[a] Age at diagnosis.

[b] Four cases with 0 survival months were excluded in this analysis.

[c] No cases in this subset in SCC patients.

[d] Only two cases in this subset and they were excluded in this analysis.
